# Fuzziness-based active learning framework to enhance hyperspectral image classification performance for discriminative and generative classifiers

**DOI:** 10.1371/journal.pone.0188996

**Published:** 2018-01-05

**Authors:** Muhammad Ahmad, Stanislav Protasov, Adil Mehmood Khan, Rasheed Hussain, Asad Masood Khattak, Wajahat Ali Khan

**Affiliations:** 1 Machine Learning and Knowledge Representation (MlKr) Lab, Institute of Robotics, Innopolis University, Innopolis, 420500, Kazan, Tatarstan, Russia; 2 Institute of Information Systems, Innopolis University, Innopolis, 420500, Kazan, Tatarstan, Russia; 3 College of Technological Innovation, Zayed University, 144534, Abu Dhabi Campus, United Arab Emirates (UAE); 4 Ubiquitous Computing Lab, Department of Computer Engineering, Kyung Hee University, Global Campus, 1 Seocheon-dong, Giheung-gu, Yongin-si, Gyeonggi-do 446-701, South Korea; PLOS, UNITED KINGDOM

## Abstract

Hyperspectral image classification with a limited number of training samples without loss of accuracy is desirable, as collecting such data is often expensive and time-consuming. However, classifiers trained with limited samples usually end up with a large generalization error. To overcome the said problem, we propose a fuzziness-based active learning framework (*FALF*), in which we implement the idea of selecting optimal training samples to enhance generalization performance for two different kinds of classifiers, *discriminative* and *generative* (*e*.*g*. *SVM* and *KNN*). The optimal samples are selected by first estimating the boundary of each class and then calculating the fuzziness-based distance between each sample and the estimated class boundaries. Those samples that are at smaller distances from the boundaries and have higher fuzziness are chosen as target candidates for the training set. Through detailed experimentation on three publically available datasets, we showed that when trained with the proposed sample selection framework, both classifiers achieved higher classification accuracy and lower processing time with the small amount of training data as opposed to the case where the training samples were selected randomly. Our experiments demonstrate the effectiveness of our proposed method, which equates favorably with the state-of-the-art methods.

## Introduction

Remote sensing is a mature field of science and extensively studied to extract the meaningful information from earth surface or objects of interest based on their radiance acquired by the given sensors at short or medium distance [1] [2]. One of the types of remote sensing is hyperspectral sensing also referred to as “hyperspectral imaging”. Hyperspectral imaging has been widely employed in real life applications such as pharmaceutical and food process for quality control and monitoring, forensic (Ink mismatches detection or segmentation in forensic document analysis), industrial, biomedical, and biometric applications such as face detection and recognition [3]. Additionally, in recent years, hyperspectral imaging has also been studied in a wide range of urban, environmental, mineral exploration, and security-related applications.

Nowadays, researchers are broadly studying hyperspectral image classification techniques for the case of a limited number of training samples, both with and without reducing the dimensionality of hyperspectral data. In this regard, the recent works [4] [5] [6] [7] [8] demonstrate that the choice of classification approach is important future research direction. Therefore, we discuss some of the main supervised and semi-supervised hyperspectral image classification techniques and their challenges.

Supervised learning techniques, which require class label information, have been widely studied for hyperspectral image classification [9]; however, these learning models face various challenges for hyperspectral image classification including but not limited to, high dimensionality of hyperspectral data and an insufficient number of labeled training samples for learning the Model [3] [10]. Collecting a large number of labeled training samples is time-intensive, challenging, and expensive because the labels of training samples are selected through human-machine interaction [11].

To cope with the issues that are discussed above, several techniques have been developed. These include discriminant analysis algorithms with different discriminant functions (e.g. nearest neighbor, linear and nonlinear functions) [12] [13], feature-mining [14], decision trees, and subspace-nature approaches [15]. The goal of subspace-nature and feature-mining approaches is to reduce the high dimensionality of hyperspectral data to better utilize the limited accessibility of the labeled training samples. The main problem of discriminant analysis is its sensitivity to the “Hughes phenomenon” [16]. The kernel based methods, like support vector machines (SVMs), have also been used to deal with the Hughes phenomenon or curse of dimensionality [17] [18] [19].

To some extent, semi-supervised approaches have addressed the problem of a limited number of labeled training samples by generating the labels though machine-machine interaction. The primary assumption of semi-supervised classification methods is that the newly labeled samples for learning can be generated with a certain degree of confidence from a set of limited available labeled training samples without considerable cost and efforts [20] [21]. Semi-supervised techniques have been significantly improved in recent years. For example, in [22] Bruzzone et al. proposed “transductive SVMs”, in [23], Camps-Valls et al. proposed a “graph-based method to exploit the importance of labeled training samples” and in [24], Velasco-Forero et al. proposed a “composite kernel in graph-based classification method”. In [25], Tuia et al. proposed a “semi-supervised SVM using cluster kernels method”, whereas in [26], Li et al. explained a “semi-supervised approach which uses a spatial-multi-level logistic prior method”. In [27], Bruzzone et al. proposed a “context sensitive semi-supervised SVM method” and Munoz-Mari et al. presented two semi-supervised single-class SVM methods in [28]. Their first technique models the data marginal distribution with graph-Laplacian built with both labeled and unlabeled training samples, whereas the other technique is used for the modification of the SVM cost function, which massively penalizes the errors made when wrongly classifying the samples for the target class. The algorithm proposed in [29] is based on a sample selection bias problem in contrast to [29], [30] where the authors proposed an SVM with a linear combination of two kernels (likelihood and base kernels). The works [31] and [32] done by Rattle, et al. and Munoz-Mari et al. respectively, exploited a similar concept using a neural network as the baseline classification algorithm. To generate the land-cover maps, they adopted a semi-automatic technique using active queries concept.

All the techniques discussed above assume that the labeled training samples are limited in number, and these methods enlarge the initial training set by efficiently exploiting the unlabeled samples to address the “ill-posed problem”. However, to achieve the desired results, several vital requirements need to be met. For example, the quantity of the generated data should not be too large such that it may increase the computational complexity, and the samples should be properly selected to avoid any confusion in correctly classifying the unseen samples. Above all, the obtained samples and their class labels must be obtained without substantial cost and time.

Active learning techniques can be used to overcome the above-mentioned issues. In general, active learning techniques are referred to as a special subcategory of semi-supervised learning techniques [33] [34]. Without loss of generality, in active learning, the learning model actively requests the user for class information. To this end, the most recent developments are “hybrid active-learning [35]” and “active learning in a single pass-context [36]”, which combine the concepts of adaptive and incremental learning from the field of traditional and online machine-learning. These breakthroughs have resulted in a significant number of different active-learning methods such as reported in [11] [26] [33] [36] [37] [38] [49] [50] [51] [52] [53].

In general, the additional labeled samples are selected randomly or by using some information criteria or source of information to query the samples and their class information. Random selection of the training samples is more-often subjective and tends to bring redundancy into the classifiers. Furthermore, it reduces the generalization performance of the classifiers. Moreover, the number of samples required to learn a model can be much lower than the number of used samples. In such scenarios, there is a risk that the learning model may get overwhelmed because of the uninformative samples queried by the learning model.

To this end, in this work, an active learning framework using a single sample view critical-class-oriented query is proposed for hyperspectral image classification. We call this scheme fuzziness-based active learning framework (*FALF*). In *FALF*, the classifier comes with an integrated data acquisition module that ranks unlabeled samples based on their confidence for the future query that has the maximum learning utility. Thus, the proposed framework aims to achieve the maximum potential of the learning model using both labeled and unlabeled data, whereas the amount of training data can be kept to a minimum by focusing only on the most informative training samples. This process leads to a better utilization of information in the data, while considerably minimizing the cost of labeled data collection and improving the generalization performance of the classifiers.

The primary goal of *FALF* is to focus on selecting difficult samples for the hyperspectral classification task. In conjunction with *“Discriminative”* and *“Generative”* classifiers, hardly predicted sample pairs are first identified by using the instability of classification boundary. A category level guidance for which sample should be queried next is then provided to the active querier. Samples with higher fuzziness and lower distance to the class boundaries are considered as the difficult samples and are queried first. This strategy of identifying the most informative samples is based on the hypothesis that, the samples that are far from class boundaries have a lower risk of being misclassified as compared to the samples that are closer. Moreover, two selection approaches are implemented and compared. The first approach randomly selects samples based on their entire fuzziness magnitude; whereas the second approach incorporates only the hardly predicted samples from higher fuzziness magnitude group.

These methods are developed for a single sample-based critical class query strategy. The experiments are conducted on both AVIRIS and ROSIS-03 hyperspectral data sets. Classification performance was superior to the state-of-the-art active learning methods. It is worth mentioning that the proposed framework is a two-fold process in which learning is first done in a fully supervised fashion, and then semi-supervised learning is used to select the appropriate candidates for the training set. Furthermore, traditional active learning methodologies add new samples to the training data with their original labels, whereas in the proposed framework, the new samples are added in a semi-supervised fashion with their predicted class labels.

To summarize, the primary contributions of our work are as follows:

- Designing and implementing a new fuzziness-based active learning framework to select the optimal training samples to enhance the classifier’s generalization performance for hyperspectral image classification.
- Validation of the effectiveness of the proposed framework for two different kinds of classifiers on three publicly available datasets (both AVIRIS and ROSIS-03 datasets).
- Investigating the potential of the proposed framework to reduce the classification time while maintaining a good accuracy under high dimensionality.

## Materials and methods

The main idea of this work is to employ and retain the relationship between misclassification rate of boundary samples and fuzziness for each class to select samples for the training set. The important steps of our proposed algorithm are summarized below:

Randomly select 5% of labeled training samples from each class.
Train Support Vector Machine (SVM) and Fuzzy K-Nearest Neighbor (FKNN) on randomly selected samples and test them for the rest of the samples.
Record the fuzzy membership matrix.
Calculate the fuzziness from fuzzy membership matrix for each sample and estimate the distance between the sample and the boundary.
Based on the threshold of fuzziness magnitude, divide the samples into two subgroups as lower and higher fuzziness magnitude groups.
Determine the correct rate of classification and misclassification (*i*.*e*., *TP and FP*) for each class in both groups individually.
(A): Pick 5% of the hardest correctly predicted samples, i.e. the samples with higher fuzziness and lower distance to the boundary. OR (B): Randomly select 5% of the correctly predicted samples, without taking into account their fuzziness and distance from the class boundary. (*Step 5*), i.e. the samples with high/lower fuzziness and lower/higher distance to the boundary.
Retrain the classifiers after adding the selected samples back into the original training set using step 7 (A) or (B), individually, and predict the rest of the samples and determine the accuracy respectively.

It is to be noted that step 7 (A) and (B) are two alternative ways to select the samples. In general active learning approaches, the samples are selected through step 7 (B), but we propose to select the samples using step 7 (A) and compare the accuracies obtained by both ways in experimental and results section.

The intuition behind selecting the hardest correctly predicted samples using step 7 (A) is that such samples contain the most information about boundaries rather than the samples with lower fuzziness in magnitude. The threshold value between lower and higher fuzziness is set by trial and error. The proposed methodology significantly boosts the performance of the classifier for hyperspectral image classification not only in terms of accuracies but also reduce the classification time.

Here we will theoretically explain the procedure for estimating the boundary of each class, and then how to build a relation between the samples and the estimated boundaries to select the target samples.

### Boundary extraction

Generally, there are two kinds of classifiers: those that use some specific formula to estimate class boundaries (discriminative), and others that use some distribution for the same task (generative). For example, [39] [40] [41] used locus approximation on some sample distributions to estimate the class boundary; whereas [42] used an analytical formula. Fuzzy K-Nearest Neighbors (FKNN’s) and Support Vector Machines (SVM’s) are two representatives of the aforementioned types.

Before we discuss the boundary extraction process for both classifiers, it is helpful to understand the concept of a fuzzy membership function, because we seek the output of each classifier in the form of fuzzy membership grades.

### Memberships function

Let us assume a set of *N* sample vectors {*r*_1_, *r*_2_, *r*_3_, …, *r*_*N*_}, and a fuzzy partition of these *N* sample vectors represents each sample vector’s degree of membership to each of the *C* classes. The fuzzy *C* partitions have certain characteristics as defined below:

$$\begin{array}{c} \sum_{\left(i=1\right)}^{C}\mu_{ij}=1 \end{array}$$

and

$$\begin{array}{c} 0<\sum_{\left(j=1\right)}^{N}\mu_{ij}<N, \end{array}$$

where *μ*_*ij*_ ∈ [0, 1], and *μ*_*ij*_ = *μ*_*i*_(*r*_*j*_) is a function that represents the membership (*a value in* [0, 1]) for the *j*^*th*^ sample *r*_*j*_, to the *i*^*th*^ partition, *i* ∈ 1, 2, 3, …, *C*, and *j* ∈ 1, 2, 3, …, *N*.

### Support Vector Machine (SVM)

SVM aims to find the optimal hyperplane according to the maximization of the margin on the training data. In SVM, data is mapped from the input space into a high dimensional feature space using an implicit function; such mapping is directly associated with a kernel function $K(r_{i},r_{j})$, which satisfies $K\left(r_{i},r_{j}\right)=\;<\varphi \left(r_{i}\right),\varphi \left(r_{j}\right)>$. In the kernel function the terms *r*_*i*_ and *r*_*j*_ denotes the *i*^*th*^ and *j*^*th*^ training samples respectively. The mathematical hypothesis of SVM is given by:

$$\begin{array}{c} f\left(r\right)=sign(\sum_{i=1}^{N}\alpha_{i}\;c_{i}\;K(r_{i},r_{j})+b) \end{array}$$ (1)

In above equation *c*_*i*_ is the *i*^*th*^ class label, b and *α*_*i*_ are unknown parameters which are determined by quadratic programming. Furthermore, *α*_*i*_ is a vector of non-negative Lagrange multipliers; therefore, the solution vector *α*_*i*_ is sparse and the samples *r*_*i*_ which correspond to nonzero *α*_*i*_ are called support vectors. Thus, the samples *r*_*i*_ corresponding to *α*_*i*_ = 0 have no contribution to the construction of the optimal hyperplane. From the literature, one can find several extensions of SVM [42] and open tools such as LIBSVM [43] which has produced acceptable performance in hyperspectral image classification. As we explained, SVM has limitations in training using a large number of samples in terms of time and computations. In order to cope with these difficulties, we can take advantage of fuzzy class membership to filter the samples based on fuzziness magnitude. In this work, we use the class membership as expressed in [44].

### Fuzzy K-Nearest Neighbors (FKNN)

FKNN produces the output as a vector of class memberships where each component of the sample vector strictly belongs to the closed interval [0, 1]. If the component of a sample vector is equal to 0 or 1, then the algorithm behaves like a common KNN. FKNN search is similar to the traditional KNN search. In traditional KNN, each sample can only belong to one class, which is the majority class in KNN search, whereas in FKNN, a sample can belong to multiple classes with different membership degrees associated with these classes. FKNN can be summarized in the following steps:

1. First find *K* nearest neighbors *r*_*j*_, *j* ∈ 1, 2, 3, …., *K*, of the given sample *r* using Euclidean distance function from the set of the samples.
2. Evaluate the membership function values for each class. FKNN obtains the membership of a sample as:
  $$\begin{array}{c} \mu_{i}\left(r\right)=\frac{\sum_{j=1}^{K}\mu_{i}\left(r_{j}\right){∥r-r_{j}∥}^{\frac{-2}{\left(m-1\right)}}}{\sum_{j=1}^{K}{∥r-r_{j}∥}^{\frac{-2}{\left(m-1\right)}}} \end{array}$$ (2)
  In the above equation ‖*r* − *r*_*j*_‖ is the Euclidean distance and *μ*_*i*_(*r*_*j*_) is the membership value of the point *r*_*j*_ for the *i*^*th*^ class. The parameter *m* controls the effective magnitude of the distance of the prototype neighbors from the sample under process [40]. The value of *m* can also be updated through cross-validation along with the value of *K*, where *K* is the number of neighbors.
3. The class of sample *r*_*j*_ is chosen by the given formula:
  $$\begin{array}{c} C\left(r\right)=argmax_{i}\left(\mu_{i}\left(r\right)\right) \end{array}$$ (3)
  where *C* is the total number of classes; therefore the decision boundary is locus expressed by (4), where $\mu_{i}^{*}$ is the permutation of *μ*_*i*_ in decreasing order.
  $$\begin{array}{c} r|\mu_{\left(i,...,C\right)}^{*}\left(r\right)=\mu_{\left(j,...,C\right)}^{*}\left(r\right),i\neq j \end{array}$$
  $$\begin{array}{c} r|\mu_{i}\left(r\right)=\mu_{j}\left(r\right)=0.5 \end{array}$$ (4)

Based on the two different classifiers’ boundary extraction process as discussed above, we can conclude that the estimated class boundary strongly depends on the criteria of classification algorithm even for identical training samples. The discussion of FKNN indicates that the boundary extraction process cannot be explicitly expressed the same way as that of the formula-based classification methods, such as SVM. Thus, it is not easy to identify whether a training sample is far from or close to the classification boundary, especially when the classification boundary cannot be expressed as a mathematical formula. Therefore, the difference between the actual and found classification boundary is considered as an important index for evaluating the generalization capability of any classification technique.

In this work, we initialize the learning model with a specific percentage of randomly selected samples, but one can control the size of samples by adjusting neighbors when assigning fuzzy class memberships to the training samples *r*_*i*_; therefore, the training set is mapped to a fuzzy training sample as (*r*_(1,….,*N*)_, *l*_(1,…,*N*)_, *μ*_(1,….,*N*)_), where each membership value is assigned independently.

### Fuzziness relation between the samples and boundary

In the above sections, we describe the process to extract the boundary of each class, but now the problem at hand is how to identify whether the said sample is close or away from the class boundary. In order to cope with the said problem, let us assume the output of a classifier for a specific sample is a fuzzy vector $\mu_{\left(1,2,3,...,n\right)}^{T}$ in which the component should be a specific number within the closed interval [0, 1]. The set of these numbers represent the fuzzy membership grades of the individual sample fit into the corresponding class.

For readers’ ease, let us consider the distance between the class boundary and the sample with output (*μ*_*i*_, *μ*_*j*_)^*T*^ can be estimated using Eq (5), which we will further incorporate with fuzziness properties. The said phenomenon is explained in the form of the corollary;

$$\begin{array}{c} |\mu_{i}-0.5|+|\mu_{j}-0.5| \end{array}$$ (5)

However, for better understanding, it is important to explain the concept of fuzziness.

#### Fuzziness properties

Consider a mapping *R* to the closed interval [0, 1] which is a fuzzy set on *R* and the mapping is denoted as *F(R)*. The fuzziness from the fuzzy membership set can be calculated as *E*: *F*(*R*) → [0, 1] or generally it can be expressed as *E*: [0, 1]^*R*^ → *R*^+^, which satisfies the following axioms as defined in [39] [45] [46]:

1. *E*(*μ*) = 0 if and only if *μ* is a crisp set,
2. *E*(*μ*) attains its maximum value if and only if *μ*(*r*) = 0.5 for all *r* ∈ *R*,
3. if *μ* ≼ *σ* then *E*(*μ*) ≽ *E*(*σ*), where *μ* ≼ *σ* ⇔ *min*(0.5, *μ*(*r*)) ≥ *min*(0.5, *σ*(*r*) and *max*(0.5, *μ*(*r*)) ≤ *max*(0.5, *σ*(*r*),
4. $E\left(\mu \right)=E\left(\bar{\mu }\right)$ where *E*(*μ*) = 1 = *μ*(*r*), and
5. *E*(*μ* ∪ *σ*) + *E*(*μ* ∩ *σ*) = *E*(*μ*) + *E*(*σ*)

Axiom 3 is known as sharpened order, where *μ* and *σ* are fuzzy subsets of a crisp set, where *μ* ≼ *σ* means *μ* is less sharpened than *σ* and hence *μ* has more fuzziness than *σ*. Since *F(R)* is not totally ordered, so there are many pairs of fuzzy sets that are not comparable under ≼ but on the contrary, a measure of fuzziness provides a total order.

#### Measuring fuzziness

Consider a fuzzy set *R* = {*μ*_(1,2,3,…,*n*)_}, then the fuzziness of *R* can be define as,

$$\begin{array}{c} \mu \left(R\right)=\mu_{i}log\left(\mu \right)+\left(1-\mu_{i}\right)log\left(1-\mu_{i}\right) \end{array}$$

$$\begin{array}{c} E\left(R\right)=\frac{-1}{n}\sum_{i=1}^{n}\mu_{i}\left(R\right) \end{array}$$

The above expression attains its maximum when the membership degree of each sample is equal to 0.5 and minimum when every sample absolutely falls into the fuzzy set or not. In this work, the term fuzziness is a kind of cognitive uncertainty.

We further extend it as a fuzzy partition of the given training samples ${\left(r_{i}\right)}_{i=1}^{P},P\ll N$ that assigns the membership degree of each sample to *C* classes as *M* = (*μ*_*ij*_)_C*P_, where *μ*_*ij*_ = *μ*_*i*_(*r*_*j*_) is the membership of the *j*^*th*^ sample *r*_*j*_ belonging to the *i*^*th*^ class. The elements of the membership matrix should follow the properties defined in the above section. Therefore, the membership matrix upon *P* training samples is attained once the training procedure completes. For the *j*^*th*^ sample, the classifier produces an output vector represented as a fuzzy set (*μ*_*j*_ = *μ*_1*j*_, *μ*_2*j*,_ *μ*_3*j*_, *μ*_*Cj*_)^*T*^, so by the above equation the fuzziness of the classifier can be written as:

$$\begin{array}{c} E\left(\mu_{j}\right)=\frac{-1}{C}\sum_{i=1}^{C}\left[\mu_{ij}log\left(\mu_{ij}\right)+\left(1-\mu_{ij}\right)log\left(1-\mu_{ij}\right)\right] \end{array}$$

Finally, a membership matrix upon *P* training samples for *C* classes can be defined as:

$$\begin{array}{c} E\left(M\right)=\frac{-1}{CP}\sum_{i=1}^{C}\sum_{j=1}^{P}[\mu_{ij}log\left(\mu_{ij}\right)+\left(1-\mu_{ij}\right)log\left(1-\mu_{ij}\right)] \end{array}$$ (6)

The above expression defines the training fuzziness. In hyperspectral space, a classifier’s fuzziness is computed as the averaged fuzziness over the entire hyperspectral space. However, the fuzziness for the testing phase is unknown. For any supervised and semi-supervised classification problem, there is a premise, “the training samples have a distribution identical to the distribution of samples in the entire space”. Therefore, the above equation can be used to calculate a classifier’s fuzziness. The following corollary gives further insight into the fuzziness relation between samples and boundary.

#### Corollary

Suppose a binary class problem with two samples (*r*_*i*_, *r*_*j*_) and distances (*d*_*i*_, *d*_*j*_), where *d*_*i*_ is the distance between classification boundary *r*_*i*_ and sample, and *d*_*j*_ is the distance between boundary *r*_*j*_ and sample.

**Fig 1:**
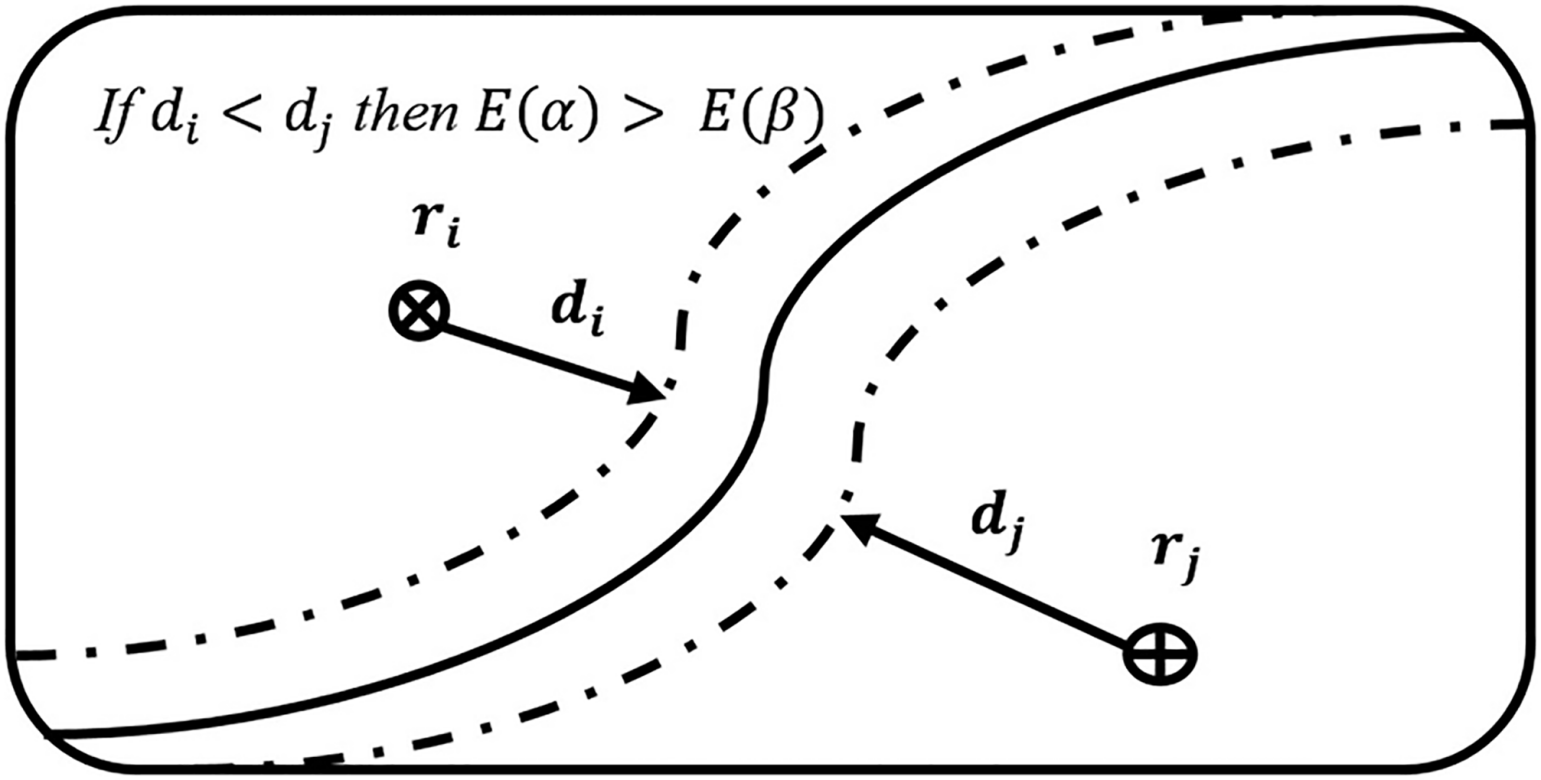
Sample distance from boundary.

Furthermore, *α* and *β* are outputs of the classifier on samples *r*_*i*_ and *r*_*j*_. According to [39], if *d*_*i*_ is less than *d*_*j*_, then the fuzziness of *α* should be greater than *β*; which means that the fuzziness of *r*_*i*_ is no less than that of *r*_*j*_. The said phenomena is further explained in Fig 1.

To prove the statement, let us assume that the outputs of the classifier on *r*_*i*_ and *r*_*j*_ are in the form of *α* = (*α*_1_, *α*_2_)^*T*^ and *β* = (*β*_1_, *β*_2_)^*T*^, respectively. Therefore, by Eqs (4) and (5), the boundary and the distance between boundary and sample with the output (*α*_1_, *α*_2_)^*T*^ can be estimated as *r*∣*α*_1_(*r*) = *α*_2_(*r*) = 0.5 and |*α*_1_ − 0.5| + |*α*_2_ − 0.5|. By the above definition, we can find the distances for each membership value as:

$$\begin{array}{c} d_{i}=|\alpha_{1}-0.5|+|\alpha_{2}-0.5| \end{array}$$

$$\begin{array}{c} d_{j}=|\beta_{1}-0.5|+|\beta_{2}-0.5| \end{array}$$

In addition, by using the above relations, we can further observe the right threshold value to identify a sample either close to or away from the boundary. Let us assume that *α*_1_ ≥ *α*_2_ and *β*_1_ ≥ *β*_2_. This implies that *α*_1_ ≥ 0.5 and *β*_1_ ≥ 0.5, which results in *d*_*i*_ = 2(*α*_1_ − 0.5) and *d*_*j*_ = 2(*β*_1_ − 0.5). Based on our assumption, *d*_*i*_ is less than *d*_*j*_, therefore *α*_1_ < *β*_1_. Thus, the sharpened order axiom *α*_1_ ≼ *β*_1_ satisfies the inequality of fuzziness as *E*(*α*_1_) > *E*(*β*_1_) but by definition, we know that *E*(*α*_1_) ≥ *E*(*α*_2_) and *E*(*β*_1_) ≥ *E*(*β*_2_), therefore, we can conclude that, *E*(*α*) > *E*(*β*).

The above mathematical evidence shows that the samples far from the classification boundary have low fuzziness as compared to the samples that are near to the classification boundary. This phenomenon is relatively simple and it is easy in a binary class problem with linearly separable samples to judge for each sample whether it is near to or away from classification boundary with some threshold value. The problem becomes trickier in case of complex boundaries with nonlinear mixtures. In such situations, we have three possibilities:

- The samples actually belong to the region where they are supposed to be; with high or low fuzziness,
- The samples belong to the other region where they are not supposed to be; with high or low fuzziness, and
- Homogeneous mixtures, i.e. non-distinguishable regions without any prerequisite conditions to make them distinguishable.

The first two cases belong to heterogeneous-type mixtures, and can easily be solved, but the third case is trickier. To cope with the third case, we suggest measuring the correct rate of classification and misclassification from each class while considering the fuzziness subgroups. This can also be solved by applying any filter, which will recursively pass the same distribution of samples at once based on their class.

## System validation

Hyperspectral image classification with an optimal number of labeled training samples is one of the fundamental and challenging tasks. In practice, the availability of labeled training samples is often insufficient for hyperspectral image classification, and in such scenarios, the classification methods generally either overwhelmed with uninformative samples or suffer due to the undersampling problem. Thus, in this work, we investigate the above-mentioned classifiers performance as a function of a different number of training samples size, varying from a minimum of 5% to a maximum of 25% per class (i.e., 5%, 10%, 15%, 20%, and 25%).

### Experimental setup

In all experiments, the parameters of the classifier are chosen as those that provide the best training accuracy. To avoid any bias, all the experiments are done within the same fixed settings which maximize the training accuracies. All the initializing parameters are evaluated in the first few experiments. When the parameters remain unchanged, the evaluation of the optimal parameters is stopped and selected for further experiments. We implemented SVM with a Polynomial kernel function and FKNN with 10 as the number of nearest neighbors and the Euclidean distance function.

In all experiments, the terms SVM and FKNN refer to the classifiers trained on samples selected using step 7(B), whereas the terms PSVM and PFKNN are used for the cases where the classifiers are trained using the samples selected using step 7(A) as explained in the methodology section. To this end, the first goal is to compare the performance of PSVM and PFKNN against that of SVM and FKNN, respectively. The second goal is to compare the performance of PSVM, PFKNN, FKNN, and SVM with state-of-the-art active learning frameworks.

The Kappa (*κ*) coefficient and overall accuracy are analyzed using a five-fold cross-validation process, related to a different number of training samples for all three datasets. It is worth noting that the training accuracy is not 100% and might include some error in terms of fuzziness estimation. All the experiments are carried out using MATLAB (2014b) on Intel^®^ Core^™^ i5 CPU 3.20 GHz with 8 GB of RAM and the Machine is the 64-bit operating system.

### Experimental datasets

The performance of the proposed FALF method is validated on three widely used publicly available hyperspectral datasets using two different classifiers with two different ways to select the target samples.

The ROSIS-03 optical sensor acquired the Pavia University (PU) and Pavia Centre (PC) data over the urban area of northern Italy. The PU and PC datasets consist of 610*340 and 1096*710 samples with 115 and 102 bands respectively. For the PU data, 12 noisy bands were removed prior to the analysis and the remaining 103 bands were used in our experiments. The ground truths differentiate 9 different classes in both datasets.

The third dataset was acquired by the Airborne Visible Infrared Imaging Spectrometer (AVIRIS) sensor. The Indian Pines (IP) dataset consists of 145*145 samples and 220 spectral bands with a spatial resolution of 20-m and a spectral range from 0.4–2.5 Î¼m. Twenty noisy bands were removed prior to the analysis whereas the remaining 200 bands were used in our experimental setup. The removed bands are 104–108, 150–163, and 220. Indian Pines dataset consists of 16 classes. All three datasets can be freely obtained from [47] [48].

### Experimental results

The Kappa (*κ*) coefficient and overall accuracies are considered as the evaluation metrics since these are widely used in existing works. The kappa coefficient is obtained by using the expressions given below [49].

$$\begin{array}{c} E\left(\kappa \right)=\frac{\left(n\sum_{k}\Psi_{k}-\sum_{k}\sigma_{k}\phi_{k}\right)}{n^{2}-\sum_{k}\sigma_{k}\phi_{k}} \end{array}$$ (7)

In the above equations, *N* is the total number of samples, *Ψ*_*k*_ represents the number of correctly predicted samples in the given class, ∑_*k*_ *Ψ*_*k*_ is the sum of the number of correctly predicted samples, *σ*_*k*_ is the actual number of samples belonging to the given class, and *φ*_*k*_ is the number of samples that have been correctly predicted into the given class [49].

**Fig 2:**
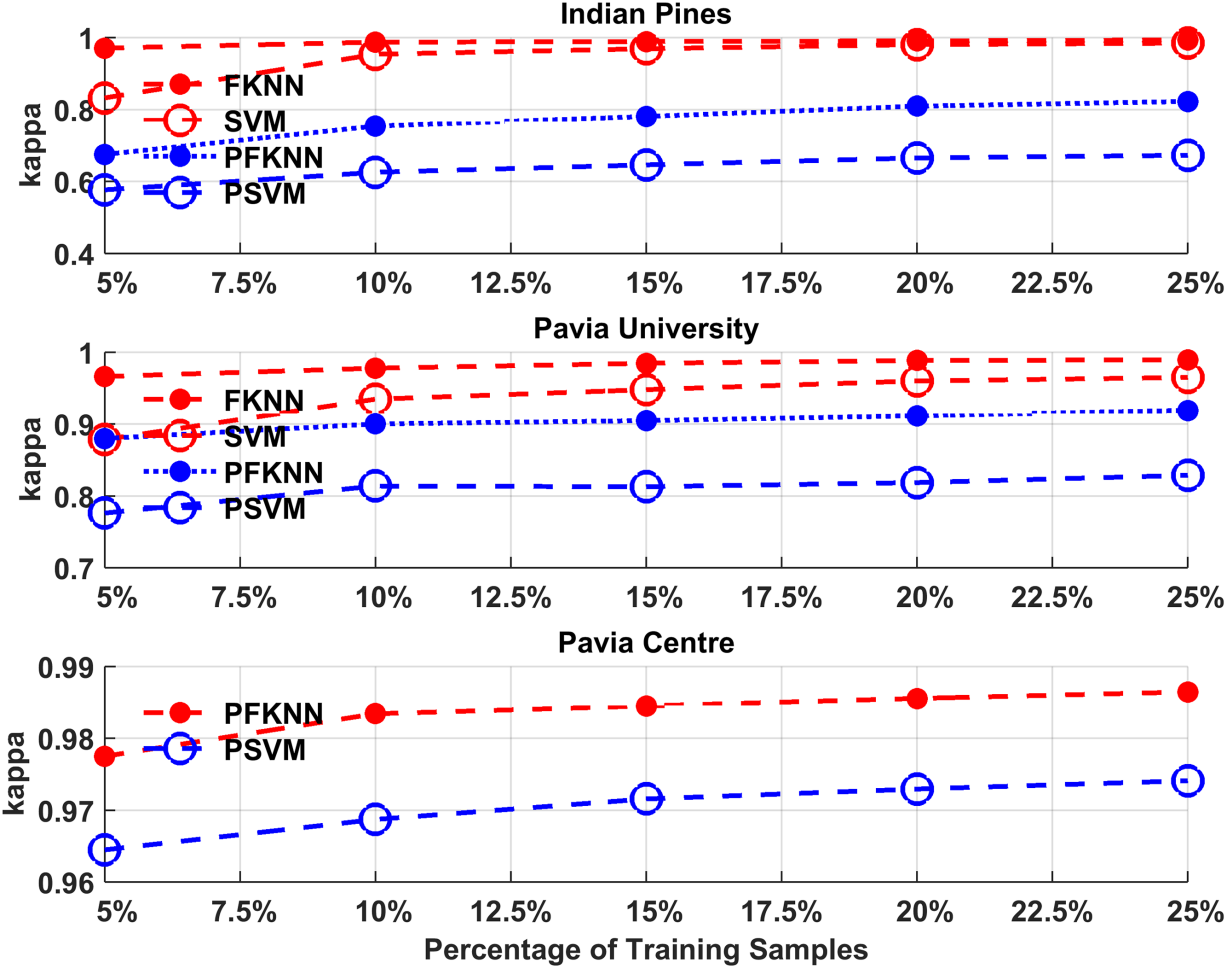
Overall classification accuracy for PSVM, SVM, PFKNN and FKNN for both Indian Pines, Pavia University, and Pavia Centre datasets.

**Fig 3:**
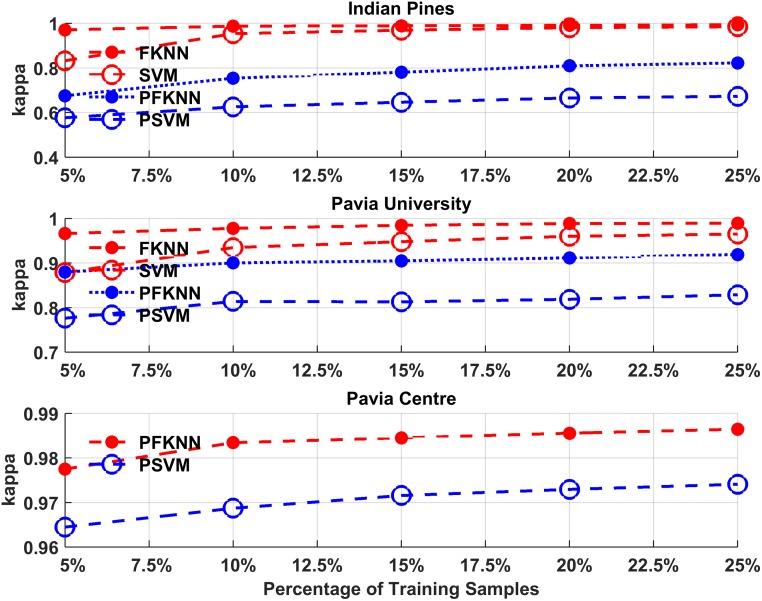
Kappa coefficient for PSVM, SVM, PFKNN and FKNN for both Indian Pines, Pavia University, and Pavia Centre datasets.

The average performance comparison of the proposed algorithm with each classifier is shown in Figs 2 and 3. These figures show the average classification accuracy and kappa coefficient analysis for both classifiers trained by randomly selected samples (Step 7(B)), and the same classifiers trained by using hardest predicted samples (Step 7(A)).

As explained earlier, we set the minimum training sample size as 5% for the first experiment and in each experiment, we increase the size with 5% newly selected samples. In the extreme case, the sample size is not more than 25% of the entire population. Based on the analysis is shown in Figs 2 and 3, for the IP and PU datasets, the PSVM classifier outperforms the rest of the classifiers. From different observations with a different number of training samples, there is a slight improvement using SVM and FKNN but PFKNN improves the accuracy impressively when we increase the size of training samples from 5% to 10% in both IP and PU datasets.

**Fig 4:**
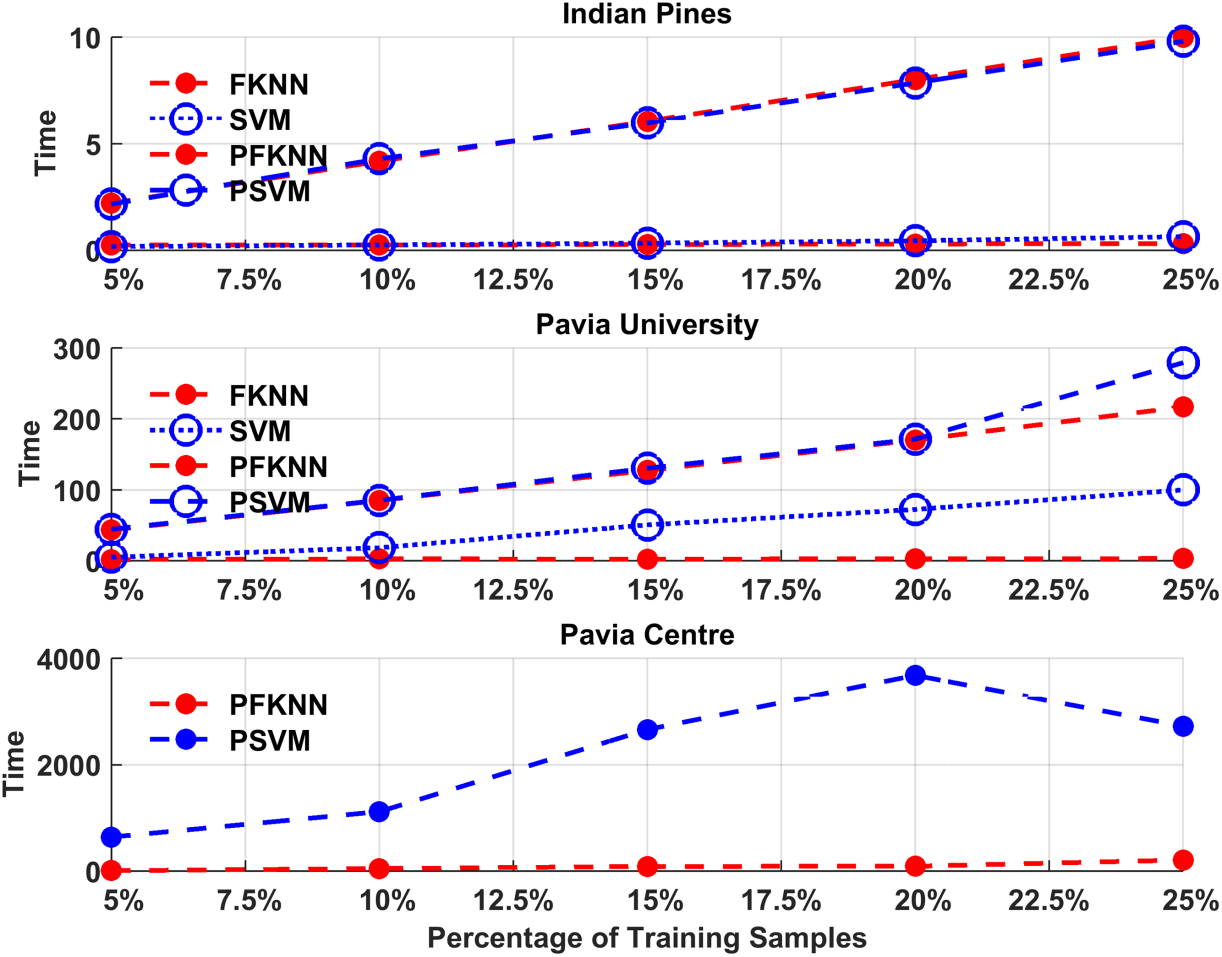
Computational time for PSVM, SVM, PFKNN and FKNN for both Indian Pines, Pavia University, and Pavia Centre datasets.

Fig 4 shows the average computational time for our first experiment. These results show that for a different number of training samples processed by PSVM, it exhibits almost identical computational cost for the same datasets, which indicates that the quantity of hardest predicted samples slightly influences the computational time of PSVM as compared to SVM. On the other hand, PFKNN obtained almost the same or a slightly lower computational time as compared to FKNN for all datasets in different experiments. In both experiments, the comparison between randomly selected samples and the hardest predicted samples has been shown for the IP and PU datasets. Moreover, the PC dataset is used to show the performance only for hardly predicted samples.

As shown in Fig 4, the computational cost gradually increases as the size of data increases in the PC dataset. Therefore, it is crucial to deal with such high computational time. Certain possible solutions can be applied to solve this problem. For example, one approach is to split the dataset into small regions and then build a separate classifier for each of the sub-regions. However, for this strategy to work well, there is another problem of how to conduct the data splitting such that it does not minimize the classification performance.

**Fig 5:**
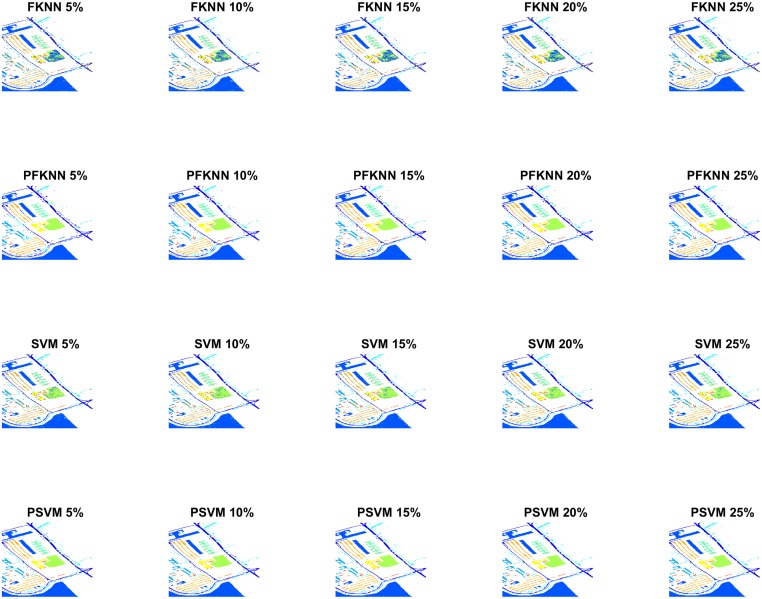
Classification maps of Pavia University (PU) with different number of training samples i.e. 5%, 10%, 15%, 20%, and 25% to train FKNN, SVM, PFKNN, and PSVM respectively.

**Fig 6:**
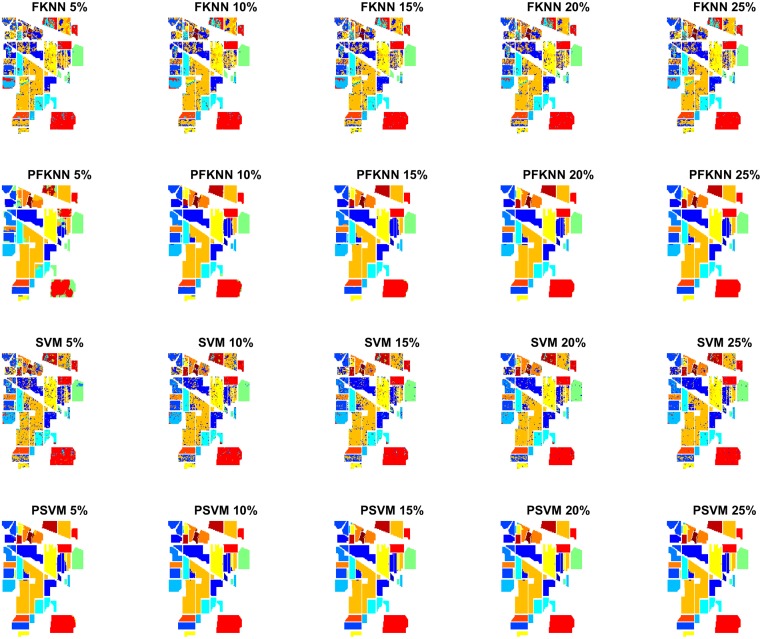
Classification maps of Indian Pines (IP) with different number of training samples i.e. 5%, 10%, 15%, 20%, and 25% to train FKNN, SVM, PFKNN, and PSVM respectively.

**Fig 7:**
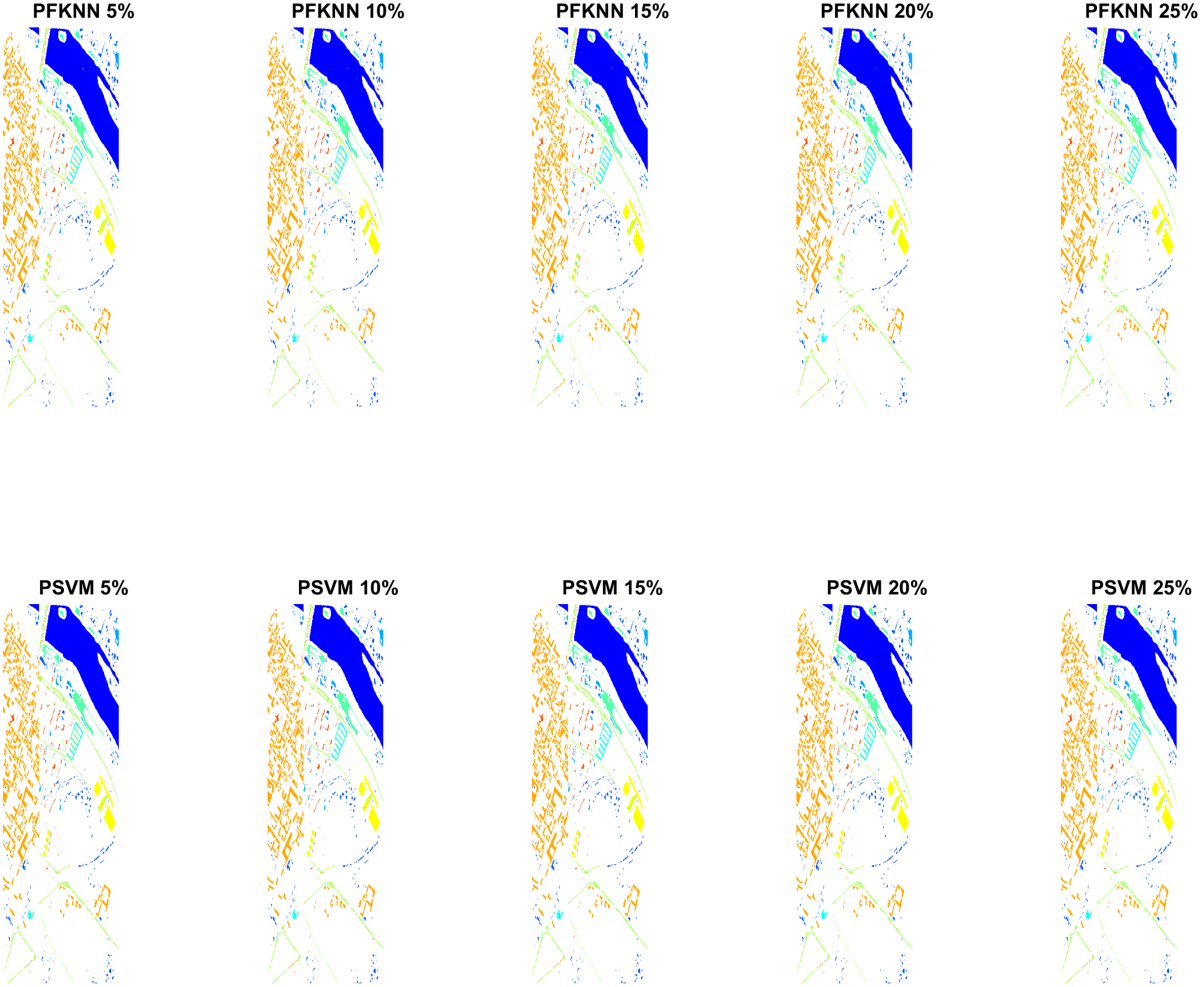
Classification maps of Pavia Centre (PC) with different number of training samples i.e. 5%, 10%, 15%, 20%, and 25% to train PFKNN, and PSVM.

Figs 5 and 6 show the classification maps for the PU and IP datasets respectively. The complete hypotheses on both datasets processed by the proposed *FALF* method to select the most informative samples to retrain the classifier, and the same classifier, trained on randomly selected samples, have been shown. Fig 7 presents the validation of our proposed model on the PC dataset. These figures show the complete performance assessment on experimental results with profound improvement. As shown in the figures, the classification maps generated by adopting the *FALF* framework are less noisy and more accurate than the maps generated by the same classifiers on randomly selected training samples.

For the trusted external judgments and for statistical analysis of any classification problem, true positive (TP), true negative (TN), false positive (FP), and false negative (FN) are usually compared. In this regard, we have made several judgments that are presented in Tables 1, 2 and 3, which show average statistics for all experimental datasets with a different number of training samples for each classifier.

**Table 1:** Indian Pines.

Classifier	PPV	FDR	FOR	LRN
**5% Training Samples**				
**FKNN**	0.47158	0.52841	0.02697	0.37514
**PFKNN**	0.74105	0.25894	0.01023	0.10667
**SVM**	0.65470	0.34529	0.02046	0.30210
**PSVM**	0.97889	0.02110	0.00184	0.03586
**10% Training Samples**				
**FKNN**	0.52308	0.47691	0.02380	0.40514
**PFKNN**	0.83876	0.16123	0.00283	0.05639
**SVM**	0.72716	0.27283	0.01560	0.22258
**PSVM**	0.98987	0.01012	0.00079	0.01493
**15% Training Samples**				
**FKNN**	0.55085	0.44914	0.02244	0.36847
**PFKNN**	0.85093	0.14906	0.00190	0.03474
**SVM**	0.75154	0.24845	0.01389	0.22821
**PSVM**	0.99135	0.00864	0.00067	0.01559
**20% Training Samples**				
**FKNN**	0.56465	0.43534	0.02129	0.24515
**PFKNN**	0.98529	0.01470	0.00120	0.01934
**SVM**	0.79040	0.20959	0.01209	0.19190
**PSVM**	0.99195	0.00804	0.00057	0.01339
**25% Training Samples**				
**FKNN**	0.59093	0.40906	0.02075	0.26579
**PFKNN**	0.98753	0.01246	0.00095	0.01565
**SVM**	0.80667	0.19332	0.01131	0.17088
**PSVM**	0.99345	0.00654	0.00040	0.00821

**Table 2:** Pavia University.

Classifier	PPV	FDR	FOR	LRN
**5% Training Samples**				
**FKNN**	0.80161	0.19838	0.02625	0.16163
**PFKNN**	0.81328	0.18671	0.01236	0.14526
**SVM**	0.87181	0.12818	0.01276	0.11241
**PSVM**	0.93518	0.06481	0.00320	0.04772
**10% Training Samples**				
**FKNN**	0.83173	0.16826	0.02161	0.14281
**PFKNN**	0.88934	0.11065	0.00642	0.08669
**SVM**	0.89917	0.10082	0.01050	0.09875
**PSVM**	0.96010	0.03989	0.00216	0.02670
**15% Training Samples**				
**FKNN**	0.82923	0.17076	0.02237	0.12797
**PFKNN**	0.90929	0.09070	0.00512	0.05990
**SVM**	0.90132	0.09867	0.00992	0.09650
**PSVM**	0.96912	0.03087	0.00149	0.02035
**20% Training Samples**				
**FKNN**	0.83511	0.16488	0.02157	0.12634
**PFKNN**	0.93043	0.06956	0.00396	0.03985
**SVM**	0.90577	0.09422	0.00919	0.09067
**PSVM**	0.97383	0.02616	0.00109	0.01718
**25% Training Samples**				
**FKNN**	0.84384	0.15615	0.02047	0.11798
**PFKNN**	0.93864	0.06135	0.00349	0.03499
**SVM**	0.91339	0.08660	0.00843	0.08203
**PSVM**	0.97341	0.02658	0.00098	0.01803

**Table 3:** Pavia Centre.

Classifier	PPV	FDR	FOR	LRN
**5% Training Samples**				
**PFKNN**	0.92469	0.07530	0.00297	0.08148
**PSVM**	0.94539	0.05460	0.00187	0.05048
**10% Training Samples**				
**PFKNN**	0.93624	0.06375	0.00261	0.07247
**PSVM**	0.96135	0.03864	0.00138	0.03626
**15% Training Samples**				
**PFKNN**	0.94072	0.05927	0.00237	0.06618
**PSVM**	0.96455	0.03544	0.00128	0.03479
**20% Training Samples**				
**PFKNN**	0.94380	0.05619	0.00225	0.06276
**PSVM**	0.96640	0.03359	0.00120	0.03250
**25% Training Samples**				
**PFKNN**	0.94339	0.05660	0.00216	0.05917
**PSVM**	0.96960	0.03039	0.00113	0.03117

We have abbreviated the test names in Tables 1, 2 and 3 as PPV = Positive Predictive Value, FDR = False Discovery Rate, FOR = False Omission Rate, and LRN = Likelihood Ratio for Negative or False Test. Positive predictive values are the scores of the positive statistical results based on TP and TN values. PPV shows the performance of a statistical measure, and we use it to confirm the probability of positive and negative results. A higher value of PPV indicates that a few positive results are a false positive.

FOR and FDR is a statistical method used in multiple hypothesis testing to correct for multiple comparisons. It measures the proportion of false negatives that are incorrectly rejected. FOR is computed by using FN and TP; it can also be computed by taking the complement of negative predictive values (NPVs). FDR measures the proportion of actual positives that are incorrectly identified and is computed by using FP and TP.

**Fig 8:**
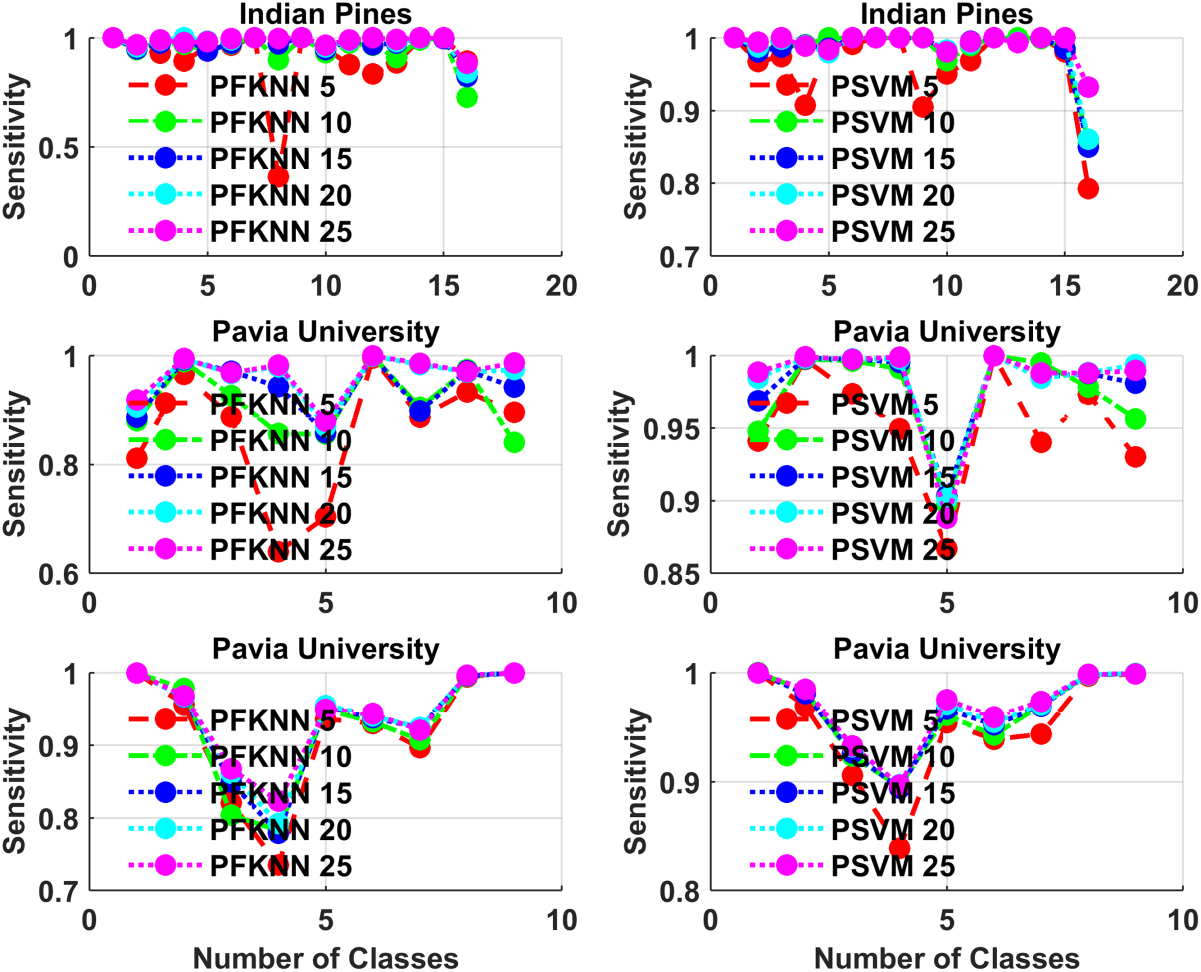
Sensitivity for each class classification analysis on Indian Pines, Pavia University, and Pavia Centre, for both PFKNN and PSVM for all three datasets.

**Fig 9:**
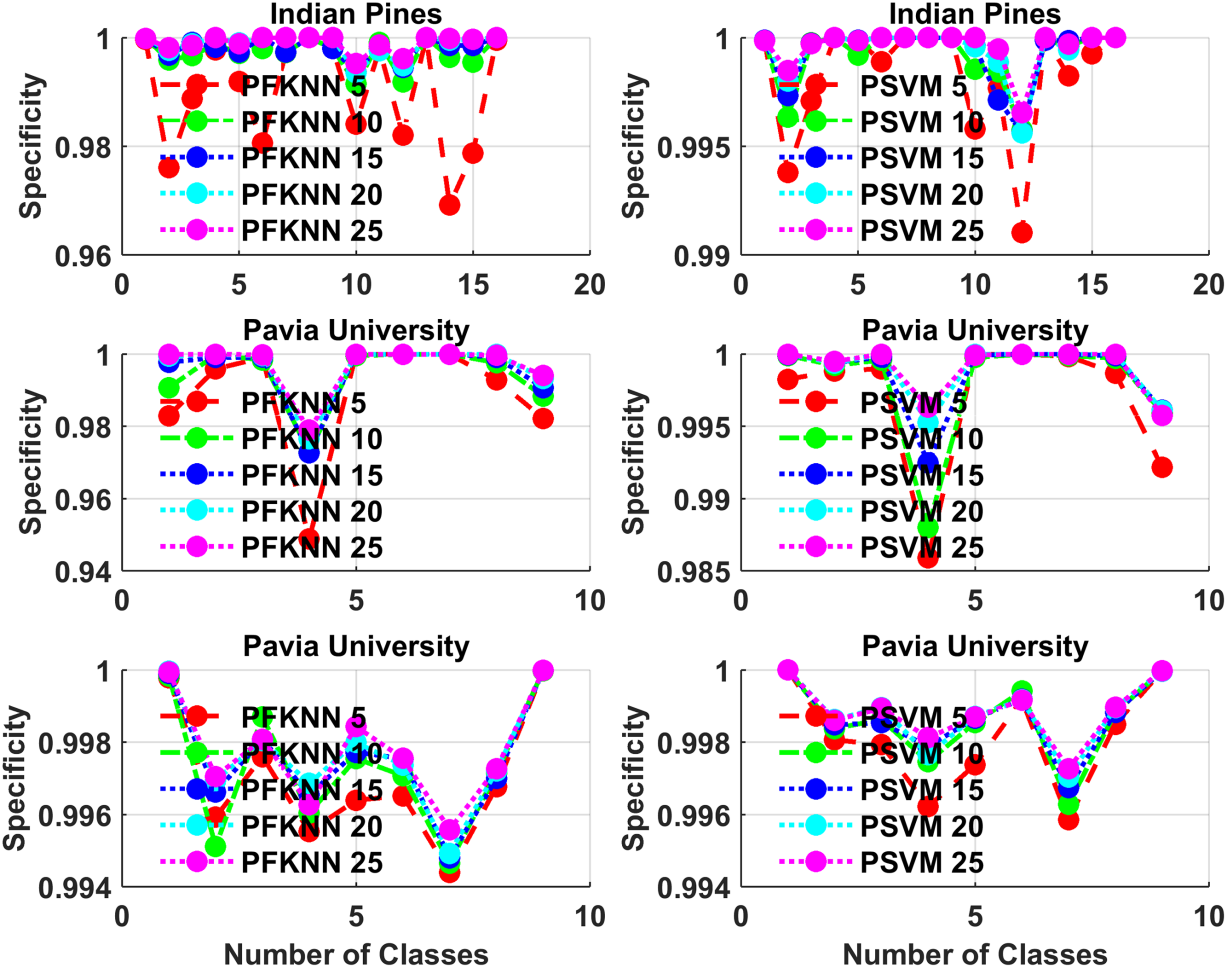
Specificity for each class classification analysis on Indian Pines, Pavia University, and Pavia Centre, for both PFKNN and PSVM for all three datasets.

For class-based classification judgments, we have done two statistical analysis, which is presented in Figs 8 and 9, in which we show the average statistics for all classes with a different number of training samples for each classifier. Figs 8 and 9 show the sensitivity and specificity of classification analysis on all three datasets for each class. PFKNN and PSVM have quite similar behavior for different classes, as one can see from the figures.

The summary classes for classification are as follows. Indian Pines: classes 1 to 16 are ““*Alfalfa*”, “*Corn Notill*”, “*Corn Mintel*”, “*Corn*”, “*Grass Pasture*”, “*Grass Trees*”, “*Grass Pasture Mowed*”, “*Hay Windrowed*”, “*Oats*”, “*Soybean Notill*”, “*Soybean Mintel*”, “*Soybean Clean*”, “*Wheat*”, “*Woods*”, “*Buildings Grass Trees Drives*” and “*Stone Steel Towers*””.

Pavia University: classes 1 to 9 are, ““*Asphalt*”, “*Meadows*”, “*Gravel*”, “*Trees*”, “*Painted Metal Sheets*”, “*Bare Soil*”, “*Bitumen*”, “*Self-Blocking Bricks*” and “*Shadows*””.

Pavia Centre: classes 1 to 9 are, ““*Water*”, “*Trees*”, “*Asphalt*”, “*Self-Blocking Bricks*”, “*Bitumen*”, “*Tiles*”, “*Shadows*”, “*Meadows*”, and “*Bare Soil*””.

## Comparison with state-of-the-art

To evaluate the performance of our proposed framework, the following state-of-the-art methods are compared. All competing methods are evaluated on two publicly available real hyperspectral datasets and the average performance of 5-fold cross validation is presented. The detailed performance comparison of the proposed algorithm with state-of-the-art methods defined below is presented in Tables 4 and 5. From these tables, we can see that the proposed framework outperforms the state-of-the-art active learning frameworks because of our careful sample selection using a twofold learning hierarchy. In traditional active learning frameworks, the supervisor selects the samples in an iterative fashion, whereas the proposed model systematically selects the samples by machine-machine interaction without involving any supervisor, in computationally efficient fashion for high-dimensional hyperspectral datasets.

**Table 4:** Indian Pines dataset.

Technique	Overall	kappa (*κ*)
**State-of-the-Art**		
SF^1^	78.14%	75.17%
SS^2^	78.78%	71.51%
SFS^3^	82.77%	80.55%
MLL^4^	**92.72**%	**91.66**%
MLL-Seg^5^	**94.76**%	**93.99**%
MLR-RS^6^	75.01%	71.49%
MLR-MI^7^	72.14%	68.27%
MLR-BT^8^	75.75%	72.27%
MLR-MBT^9^	75.73%	72.22%
OS^10^	81.68%	79.25%
RS^11^	81.54%	79.89%
MPM^12^	85.42%	83.31%
LBP^13^	**95.92%**	**95.34%**
RS^14^	85.33%	83.26%
MBT^15^	**91.98%**	**90.84%**
BT^16^	**92.16%**	**91.08%**
MI^17^	87.02%	85.23%
LORSAL^18^	82.60%	80.14%
**Proposed Framework**		
FKNN^19^	67.62%	60.57%
SVM^20^	78.51%	75.44%
PFKNN^21^	**95.94%**	**95.36%**
PSVM^22^	**98.88%**	**98.73%**

^1^Adseg-AddFeat (SF),

^2^Adseg-Addsamp (SS),

^3^3Adseg-AddFeat + AddSamp (SFS),

^4^Multilevel Logistic (MLL),

^5^Multilevel Logistic over Segmentation Maps (MLL-Seg),

^6^Multinomial Logistic Regression for Random Selection (MLL-RS),

^7^Multinomial Logistic Regression for Mutual Information (MLR-MI),

^8^Multinomial Logistic Regression for Breaking Ties (MLR-BT),

^9^Multinomial Logistic Regression for Modified Breaking Ties (MLR-MBT),

^10^Over Segmentation Maps (OS),

^11^Redefined Segmentation Maps (RS),

^12^Maximum Posteriori Marginal (MPM),

^13^Maximum Posteriori Marginal based Loopy Belief Propagation (LBP),

^14^Maximum Posteriori Marginal and Loopy Belief Propagation based Random Selection (RS),

^15^Maximum Posteriori Marginal and Loopy Belief Propagation based Modified Breaking Ties (MBT),

^16^Maximum Posteriori Marginal and Loopy Belief Propagation based Breaking Ties (BT),

^17^Maximum Posteriori Marginal and Loopy Belief Propagation based Mutual Information (MT),

^18^Logistic Regression via Variable Splitting and Augmented Lagrangian Algorithm (LORSAL),

^19^Random Selection (FKNN),

^20^Random Selection (SVM),

^21^Hardly predicted (PFKNN), and

^22^Hardly predicted (PSVM).

**Table 5:** Pavia University dataset.

Technique	Overall	kappa (*κ*)
**State-of-the-Art**		
SF^1^	90.71%	88.05%
SS^2^	86.58%	82.73%
SFS^3^	**92.23%**	**90.05%**
MLL^4^	85.57%	81.80%
MLL-Seg^5^	85.78%	82.05%
MLR-RS^6^	86.61%	82.49%
MLR-MI^7^	85.88%	81.50%
MLR-BT^8^	85.63%	81.21%
MLR-MBT^9^	85.24%	80.70%
OS^10^	**91.08%**	**91.21%**
RS^11^	**91.58%**	**91.62%**
MPM^12^	85.78%	82.05%
LBP^13^	—–%	—–%
RS^14^	**93.45%**	**91.40%**
MBT^15^	**95.85%**	**94.61%**
BT^16^	**95.80%**	**94.54%**
MI^17^	**96.86%**	**95.87%**
LORSAL^18^	**94.02%**	**92.05%**
**Proposed Framework**		
FKNN^19^	86.26%	81.38%
SVM^20^	**92.50%**	**90.04%**
PFKNN^21^	**95.94%**	**93.47%**
PSVM^22^	**98.32%**	**97.78%**

^1^Adseg-AddFeat (SF),

^2^Adseg-Addsamp (SS),

^3^3Adseg-AddFeat + AddSamp (SFS),

^4^Multilevel Logistic (MLL),

^5^Multilevel Logistic over Segmentation Maps (MLL-Seg),

^6^Multinomial Logistic Regression for Random Selection (MLL-RS),

^7^Multinomial Logistic Regression for Mutual Information (MLR-MI),

^8^Multinomial Logistic Regression for Breaking Ties (MLR-BT),

^9^Multinomial Logistic Regression for Modified Breaking Ties (MLR-MBT),

^10^Over Segmentation Maps (OS),

^11^Redefined Segmentation Maps (RS),

^12^Maximum Posteriori Marginal (MPM),

^13^Maximum Posteriori Marginal based Loopy Belief Propagation (LBP),

^14^Maximum Posteriori Marginal and Loopy Belief Propagation based Random Selection (RS),

^15^Maximum Posteriori Marginal and Loopy Belief Propagation based Modified Breaking Ties (MBT),

^16^Maximum Posteriori Marginal and Loopy Belief Propagation based Breaking Ties (BT),

^17^Maximum Posteriori Marginal and Loopy Belief Propagation based Mutual Information (MT),

^18^Logistic Regression via Variable Splitting and Augmented Lagrangian Algorithm (LORSAL),

^19^Random Selection (FKNN),

^20^Random Selection (SVM),

^21^Hardly predicted (PFKNN), and

^22^Hardly predicted (PSVM).

1. Multinomial Logistic Regression with Active Learning [26]
2. Active Learning framework using hierarchical segmentation [50]
3. Spatial Coherence Batch Mode Active Learning [51]
4. Spectral-Spatial Classification using Loopy Belief Propagation and Active Learning [52]

## Discussion

We can find many classical active learning frameworks in the literature that are similar to the proposed framework. For example, the work proposed by Lughofer in [36] focused on online learning and it was specifically designed for “an on-line single-pass setting in which the data stream samples arrive continuously”. Such kind of methods does not allow classifier re-training for the next round of sample selection. Furthermore, Lughofer uses the close concepts of conflict and ignorance. Conflict models how close a query point is to the actual decision boundary and ignorance represents the distance between a new query point and the training samples seen so far. Our membership concept is conceptually close to these indicators, but we are able to consider both the distance from the class boundary and in-class variance inside one parameter. In addition, unlike [36], we implemented and validated our active learning approach for hyperspectral image classification problem.

In contrast to [36], Nie et al. proposed another active learning framework in [53], in which the authors focused only on early active learning strategies, i.e., solving the early stage experimental design problem. The Transductive Experimental Design (TED) method was proposed to select the data points, and for this, the authors propose a novel robust active learning approach using the structured sparsity-inducing norms to relax the NP-hard objective to the convex formulation. Thus their framework only focused on selecting an optimal set of initial samples to kick-start the active learning procedure. However, the benefit of our framework is that it shows state-of-the-art performance independent of how the initial samples are selected. Of course, the framework proposed by Nie et al. can be easily integrated with our framework to be executed instead of executing the first step of our algorithm.

In our work, we start evaluating our hypotheses from 5% of randomly selected training samples and we demonstrate that randomly adding more samples (step 7(B)) back into the training set slightly increases accuracy but the classifiers become computationally complex. Therefore, we decided to separate the set of samples that were most difficult to predict in our first phase of classification (*The samples between the ranges of 0.7–1.0 in fuzziness magnitude*). We then fuse a specific percentage of these hardly predicted samples back into the original training set to retrain the classifier from scratch for better generalization and classification performance on those samples which were initially misclassified.

It is worth noting from experiments that adding hardly predicted samples back into the training set improves the performance on those samples that were misclassified in the first phase. For the IP and PU datasets, we have experimentally proved that randomly adding samples back into the training set does not provide the desired accuracy, but that by adding the samples back into the training set selected by the proposed FALF framework boosts the performance of the classifier. We further validate our hypotheses on the PC dataset which also produces good accuracy in a computationally efficient way.

A second most important factor involved in the training and testing phase is computational time, which is significantly improved for both classifiers. Therefore, to make the model efficient and quick, we fuse the most difficult and informative samples back into the training set to retrain the classifier in each experiment. The classification accuracy and Kappa (*κ*) test results are significantly improved as we can see from Figs 2 to 9. Tables 1, 2 and 3 present the average statistical test results on predicted samples, which show the model’s ability to correctly classify the unseen samples from each class.

To experimentally observe a sufficient quantity of training samples for each classifier, we evaluated the hypothesis as explained earlier. Based on the experimental results, we conclude that the 10% samples obtained by the proposed *FALF* framework are good enough to produce the acceptable accuracy for hyperspectral image classification with minimum computational cost.

## Conclusion

Hyperspectral image classification with a limited number of training samples is a challenging problem. To improve the classification performance for such cases, this paper proposed the idea of retraining the classifier using most informative samples. These samples are identified by first estimating the boundary of each class and then calculating the fuzziness-based distance between each sample and the estimated class boundaries. The hardest correctly classified samples with smaller distances and higher fuzziness are selected as appropriate candidates for the training set to retrain the classifier.

Through several experiments, we show that for an image classification task we can start with only 5% of the training samples and then use the proposed *FALF* framework to select only a small amount of new samples to train the classifier from scratch, which significantly boosts the classifier’s generalization performance on unseen samples.

It is worth noting is that the proposed method is not classifier sensitive, i.e. the derived relation holds if we change the classification model, such as locus approximation to an analytical formula-based classifier.

## Supporting information

S1 File The Indian Pines (corrected) dataset, consisting of 145*145 samples and 220 spectral bands with a spatial resolution of 20 m and a spectral range of 0.4–2.5 Î¼m. Twenty noisy bands were removed prior to the analysis, whereas the remaining 200 bands were used in our experimental setup. The removed bands are 104–108, 150–163, and 220. The original Indian Pines dataset is available online at [47] [48].
(TXT)

S2 File The original Indian Pines ground truths consist of 16 classes. The ground truth classes and the number of samples per class (class name-number of samples) are as follows: ““*Alfalfa-46*”, “*Corn Notill-1428*”, “*Corn-Mintel-830*”, “*Corn-237*”, “*Grass Pasture-483*”, “*Grass Trees-730*”, “*Grass Pasture Mowed-28*”, “*Hay Windrowed-478*”, “*Oats-20*”, “*Soybean Notill-972*”, “*Soybean Mintel-2455*”, “*Soybean Clean-593*”, “*Wheat-205*”, “*Woods-1265*”, “*Buildings Grass Trees Drives-386*” and “*Stone Steel Towers-93*””. The ground truths are freely available at [47] [48].
(TXT)

S3 File This file contains MATLAB code for reshaping and rewriting of the original Indian Pines dataset and ground truths into text files as per journal requirements. (M)
